# Building a strong collaborative biostatistics workforce: Strategies for effective intra-unit professional development activities

**DOI:** 10.1017/cts.2023.653

**Published:** 2023-10-19

**Authors:** Sandra L. Taylor, Robert H. Podolsky, Maria E. Montez-Rath, Emily Slade

**Affiliations:** 1 Department of Public Health Sciences, School of Medicine, University of California, Davis, CA, USA; 2 Division of Biostatistics and Design Methodology, Children’s National Hospital, Silver Spring, MD, USA; 3 Division of Nephrology, Department of Medicine, Stanford University School of Medicine, Stanford, CA, USA; 4 Department of Biostatistics, University of Kentucky, Lexington, KY, USA

**Keywords:** Professional development, collaboration, biostatistics, team science, career advancement

## Abstract

Ongoing professional development is important for collaborative biostatisticians, as it enables them to remain current with the latest advances in statistical methodology and software, refine their analytical skills, and expand their domain knowledge, thereby facilitating their ability to contribute effectively to biomedical research. Although external opportunities for professional development, such as attending conferences and workshops, are widely recognized and valued in the field of biostatistics, there has been comparatively little attention given to internal opportunities for enhancing the skills and knowledge of biostatisticians which can be implemented with lower financial and time investment than external offerings. The purpose of this paper is to offer guidance for ongoing internal professional development activities that can be employed by collaborative biostatistics units in universities and academic medical centers to complement structured curricula and initial training. Specific examples of activities are provided so that collaborative biostatisticians and/or managers of biostatistical units can flexibly combine components to create an appropriately scaled, customized program that meets the needs of themselves or of the unit.

## Introduction

A primary focus at academic medical centers is clinical and translational research to further human health and well-being. Rigorous and appropriate study design and statistical analyses are foundational to advance this objective. Biostatisticians with technical skills, scientific understanding, and interpersonal skills are critical for the success of medical research at these institutions [1–4]. Most university medical centers have academic departments that house faculty in biostatistics who collaborate with medical investigators [2,5]. Often staff biostatisticians at the master’s and doctoral levels fulfill a vital and complementary role to faculty in meeting the demands for statistical expertise and data analysis at these institutions [1].

A collaborative biostatistician works with teams of clinical collaborators, many of whom are required to complete continuing education to stay knowledgeable about evidence-based developments in healthcare fields [6–8]. Even though methodological and computational tools are constantly evolving, biostatisticians typically do not have credentialling requirements for continuing education as their clinical counterparts do. Despite the lack of formal regulation for continuing education for biostatisticians, it is imperative for biostatisticians to emulate their clinical counterparts to maintain a leading edge in their respective discipline and thereby facilitate optimal team science. Further, employees generally value continued learning and skill development, and opportunities for these activities can enhance job satisfaction and employee retention [9–11].

A typical approach to ongoing professional development for biostatisticians is to attend external conferences or workshops. Despite the rise of virtual meetings, attending external offerings remains expensive and time-consuming, thus limiting the number who can attend. Further, external options are sporadic and may not be specific enough to be valuable locally. One-on-one mentoring between senior and junior personnel in the conduct of specific projects is also a common and very important avenue for professional development. However, while tailored to an individual’s development needs, one-on-one mentoring is inefficient and does not offer the team building benefits of group activities. The barriers to and shortcomings of external development options and limitations of one-on-one mentoring argue for communal, home-grown professional development activities that can be tailored to specific needs of personnel (staff and faculty) in a single biostatistical unit. Moreover, substantial expertise is commonly available internally and can be leveraged.

Recognizing the need and value for ongoing professional development for biostatisticians, strategies and approaches for professional development that are cost-effective and time-efficient are essential given budget and schedule constraints [12,13]. In this paper, we propose approaches to integrating communal, ongoing learning, and development activities for a biostatistics unit. These activities are distinct from but complementary to formal training of skills necessary for collaborative biostatisticians [8]. Further, while we focus on collaborative biostatisticians, our proposed approaches are broadly applicable to other groups that bring their technical skills to collaborative research such as bioinformaticians and data scientists.

We highlight the importance of ongoing professional development for biostatisticians and emphasize fostering a culture of promoting the value of professional development for staff and faculty alike. We first touch on the skill domains important for collaborative biostatisticians. Then, we detail strategies and approaches that can be efficiently employed and integrated into normal unit operations to provide ongoing professional development. Our proposed approaches have low financial and time commitment barriers.

These approaches are illustrated with concrete examples and case studies. However, because the size, structure, needs, resources, and culture of biostatistics units are highly variable, we specifically avoid prescriptive recommendations and encourage units to develop individualized programs. Finally, we identify barriers to implementation and suggest avenues to overcome those barriers. We emphasize that professional development activities can be organized by unit managers or team leaders, or initiated directly by individuals or groups of staff/faculty who are seeking ongoing professional development as collaborative biostatisticians. The perspectives in this paper come from experiences leading collaborative biostatistics groups of various sizes and structures at four different academic medical centers.

## Focus Areas of Professional Development: Essential Skill Domains

As detailed elsewhere [8], essential skills for collaborative biostatisticians fall into three broad domains: statistical expertise, clinical and domain knowledge, and communication and leadership. Command of common analytical techniques, awareness of a broad array of statistical methods, software coding, study design options, preparation of statistical analysis plans, and practices for ensuring reproducibility fall under the statistical expertise domain. Clinical and domain knowledge encompasses scientific understanding and reasoning, familiarity with and intuition about common medical and clinical terms and variables, sensitivity to institutional structure and values, regulatory requirements, and sources and quality of commonly used data and databases. The communication and leadership domain covers written and verbal communication skills with non-statisticians, ability to present results in a meaningful way, time management, project and task management, appropriate prioritization, and ethical practices. Unit members at all levels (entry-level analysts to managers and faculty) can benefit from continued development in these skill domains [8].

## Intra-Unit Professional Development Components

**Figure 1.**
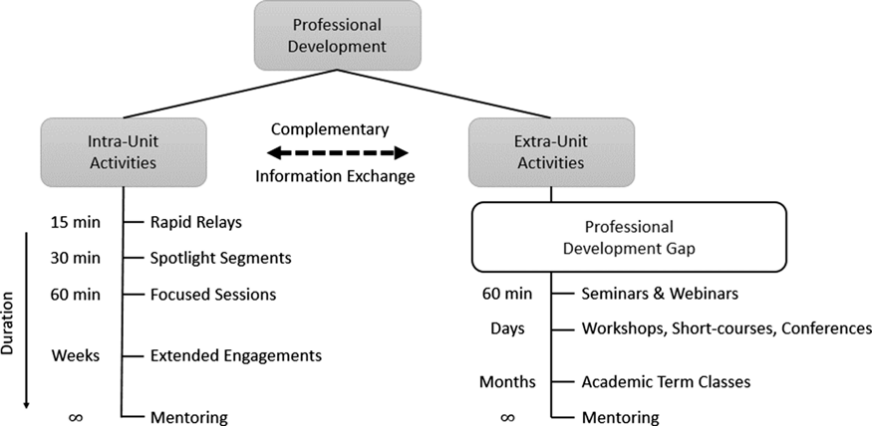
Complementary alignment of intra- and extra-unit professional development activities.

While some biostatisticians may take advantage of external professional development opportunities such as attending conferences, our focus is on collective professional development opportunities that can be implemented within a biostatistics unit and be incorporated into regular unit operations in an ongoing fashion (Fig. 1). We focus on collective intra-unit activities which we define as activities conducted by members of a biostatistical unit in contrast to extra-unit activities which are implemented by entities outside of the biostatistical unit (Fig. 1). A unit is any formally or informally defined group of collaborative biostatisticians potentially composed of any combination of staff and faculty. Our objective is to provide continual learning and idea exchange for the unit as a whole as a complement to specific training. Building blocks for a program can be organized by the duration of a particular activity, and we have grouped activities into four categories: Rapid Relays, Spotlight Segments, Focused Forums, and Extended Engagements (Table 1).

**Table 1.** Examples of specific activities for the four components for intra-unit professional development activities by the skill domains needed for being a successful collaborative biostatistician

	Setting			
Skill domains	Rapid Relays	Spotlight Segments	Focused Forums	Extended Engagements
	5–15 minutes	30 minutes	60–90 minutes	Repeated meetings over several weeks
Statistical expertise	Demonstrate making tables using the tableby() function in the *arsenal* package in R	Application of competing risk time-to-event methods for analyzing death due to cardiovascular causes where death due to non-cardiovascular causes is a competing risk	Introductory seminar on Bayesian inference	Study and application of machine learning methods for clinical prediction
Clinical and domain knowledge	How is race/ethnicity coded at our institution?	Presentation about what the IRB is and how does it relate to the work of a statistician.	A collaborating physician gives a seminar on their field of practice to enhance clinical knowledge of statisticians	Multiple study session to learn about how organ transplantation works in the USA: what are the rules, requirements, known outcomes, and data availability.
Communication and leadership	How to follow up a meeting with an email documenting next steps and tasks. How to respond to a request to meet an unreasonable deadline. How to respond to request for inappropriate statistical analysis	How to preparing a technical memorandum reporting analysis results of a longitudinal study including setting, objectives, statistical methods, and results with illustrative tables and figures.	Workshop on how to navigate difficult conversations with collaborators, such as scope of work, effort allocation, authorship, timelines, scientific integrity, and ethical concerns	Ongoing meetings where group members practice creating and delivering seminars to non-statisticians and receive feedback on presentation and delivery

### Rapid Relays (5–15 Minutes)

Rapid Relays consist of a short exchange of information spanning 5 to 15 minutes. Rapid Relays are ideal for presenting how to use a particular statistical software function, providing an overview of a statistical software package, or a similarly specific, well-defined topic. This type of professional development can be easily integrated into regular (e.g., weekly) staff meetings. Rapid Relays can be permanent items on staff meeting agendas with responsibility for preparing for a Rapid Relay rotated among unit members, thus ensuring engagement of all members at some point regardless of personality. The exact frequency of Rapid Relays will depend on the culture of and the number of biostatisticians in the unit, with less frequent Rapid Relays (e.g., monthly) in smaller units to avoid making Rapid Relay preparation burdensome. Alternatively, a Rapid Relay can be added to an upcoming agenda only when a salient topic arises. For example, unit members who attend a workshop or seminar can share something they learned through this format (Table 1). Rapid Relays offer a short, low-stakes presentation opportunity that facilitates sharing by all unit members, providing an opportunity to build confidence and enhance presentation skills for early career members.

### Spotlight Segments (∼ 30 Minutes)

Spotlight Segments are longer engagements than Rapid Relays but are still short and focused. At about 30 minutes in duration, they provide some opportunity for discussion and feedback among the group of biostatisticians. This duration is sufficient for unit members to present a challenging analysis problem and solicit input from others on potential solutions, or to present how they approached and solved a nonstandard analysis. Presentations on statistical methodology or study design with an illustrative application are accommodated in this type of activity (Table 1). A 30-minute session is also long enough for meaningful instruction or discourse on collaboration and leadership skills. Spotlight Segments can be integrated into regular staff meetings or held as separate recurring events.

### Focused Forums (60–90 Minutes)

The 60-minute seminar with one or multiple speakers is a traditional approach to professional development. While Focused Forums can be a traditional seminar delivered by intra- or extra-unit staff or faculty, they do not need to follow this format. Activities of this duration are ideal for active participation of attendees. As such, they can encompass workshops with hands-on activities for participants or brainstorming sessions. An example of a workshop includes a working session on how to write a statistical methods section for a manuscript. A brainstorming session could be a session where a member presents a challenging data analysis project and seeks input on approaches.

Professional development activities of this duration are most likely to take place separate from routine staff meetings. Staff and faculty from outside the unit can be invited to intra-unit Focused Forums, and some Forums could be developed and scheduled in cooperation with extra-unit personnel. Outside of an individual biostatistics unit, many Statistics and Biostatistics departments have a regular seminar series, and some professional societies for statisticians offer free online seminars or workshops. Intra- and extramural offerings can greatly broaden opportunities for professional development in all of three essential domains (statistical expertise, clinical and domain knowledge, and communication and leadership).

### Extended Engagements (Multiple Meetings)

For some topics, an extended course of study is necessary for biostatisticians to gain proficiency in the skill. Extended Engagements consist of a small group or an individual committing to study a particular topic over several weeks. For example, a group could focus on learning foundations and applications of Bayesian inference (Table 1). These groups can have targeted in-depth instruction sessions led by group members or faculty experts. The duration of an Extended Engagement depends on the topic, the time availability, and the commitment level of participants. These professional development activities are most likely to arise when a small group has an interest in developing expertise or improving their skills in a particular area. The group organizes itself, committing to regular meetings to learn a method or skill and practice applying it. Extended Engagement groups could include extra-unit personnel such as isolated statisticians from other departments/units.

Given the long duration and active participation requirement, Extended Engagements are not professional development activities that can be easily integrated into existing meeting structures. Managers of biostatistics staff can enhance the success and impact of these activities by allocating time to participate and providing any other resources needed as well as detailing objectives, timelines, and milestones for the activity. However, less formally, a self-directed group could regularly meet during a lunch period to minimize work impacts and would require minimal resources beyond a meeting space.

## Implementation of Professional Development Activities

We have detailed different components that can be combined for an intra-unit professional development program. Which components are used and how they are combined will depend on the professional development goals and objectives of the individual unit as well as available resources (personnel, time, and financial). In implementing a professional development program, we encourage managers and staff to take ownership of their professional development activities and work collaboratively to develop intra-unit activities such that all parties are vested in the program. We reiterate that all collaborative biostatisticians from an entry-level statistician to tenured faculty should view professional development activities as a requirement of being a professional consistent with many other careers and actively engage in program development and activities.

### Goals and Objectives for Professional Development Activities

As a first step, we suggest unit members (managers, staff, and faculty) develop a common vision of what they want to gain from professional development activities. We encourage managers to engage unit members regarding their professional development interests. Questions to consider include:

- Who wants to participate in professional development? For those who do not, why not?
- What professional development opportunities do unit members currently participate in?
- What do unit members see as the unit’s current professional development needs?
- If the unit includes faculty, are there differences in what types of professional activities staff and faculty want?
- Are there subgroups (e.g., staff and junior faculty) who desire separate activities to address unique needs?
- What knowledge, skills, and abilities do unit members want to improve or acquire?

### Resources and Environment

With goals and a shared vision for professional activities, the unit is positioned to assess the current environment, time, and financial resources and constraints. Some questions to consider are:

- How large is the team that will regularly participate in these activities?
- Do you currently meet regularly as a team? If so, how often do you meet and for how long?
- What is the typical focus of and reason for your existing team meetings?
- Who attends the team meetings? Is it staff only or are faculty members in attendance as well?
- Realistically, to what extent can schedules and workload accommodate additional or expanded meetings?
- Given the goals and needs previously identified, what components can be used to meet the goals?
- Can components (e.g., Rapid Relays and Spotlight Segments) be combined with the existing meeting structure?
- Are professional development meetings separate from administrative meetings necessary and desirable?
- Are financial resources necessary and available for any activities?
- How much time do unit members have to attend and prepare for professional development activities?

### Logistical Considerations

Several logistical issues will need to be addressed when establishing any professional development program. Below are logistical questions to consider when designing and initiating a program.

- How often do you want to meet and for how long?
- Who will be in charge of deciding on topics and leading meetings? Will leadership rotate?
- What format will be used (e.g., in person, fully remote, or hybrid)?
- What constraints or challenges are present, and how will these challenges be addressed?
- Will resources developed as part of professional development activities be stored in a central repository for later access? If so, where will they be stored?

## Assembling the Components

Once the team’s needs, preferences, challenges, and opportunities have been detailed, all unit members can be active participants and together strategize on how to build a professional development program. The answers to the above questions will drive what components are used and how they are combined. Depending on the maturity of the team and individual personalities, this may take some mentoring and encouragement from managers. In Supplemental Material, we illustrate how a program within a hypothetical unit could be initiated and matured.

Small units can establish goals and build a program through real-time discussions, but this approach may be unwieldy, unproductive, or inefficient for larger units. For larger units, surveys could be helpful in obtaining input from as many members as possible for use by a committee to develop the program. We also suggest revisiting these questions periodically as unit membership evolves. Small units may use different components than large units due to resource availability. Such units might elect to include Rapid Relays and/or Spotlight Segments as part of regularly scheduled administrative meetings, since these components can be implemented with little time and monetary cost. Larger units may choose to organize Focused Forums and Extended Engagements, since these are better suited for separate development-focused meetings and necessarily require more resources.

We provide two case studies representing a small, growing biostatistical unit (Table 2) and a large, well-established unit (Table 3) to illustrate different approaches. These case studies show how the components can be combined, customized, and implemented to best serve the respective units.

**Table 2.** Case study 1: University of California, Davis, Biostatistics Support Unit

Personnel	The Clinical and Translational Science Center Biostatistics Program at the University of California, Davis, consists of 5 MS-level biostatisticians, 3 PhD-level biostatisticians, a faculty director, and a faculty associate director. Four staff (2 MS and 2 PhD) are fully remote with the remainder working a hybrid schedule.
Goals and objectives	Continued development of technical (e.g., statistical knowledge and coding) and collaborative skills of staff. Facilitate engagement and connections among MS-level statisticians. Develop understanding of team’s collective knowledge, that is, who knows what. Increase collaboration among statisticians to improve the team’s collective statistical practice. Improve verbal communication skills.
Challenges	Master’s-level staff are reluctant to participate in discussions when faculty and, to some extent, PhD-level staff are present. Staff have both remote and hybrid work schedules. Some remote staff work in different time zones some or all of the time.
Activities in practice	Professional development activities exploit the Focused Forums, Spotlight Segments, and Rapid Relays formats. Monthly, one and a half hour-long meetings focused specifically on professional development activities are held. Local staff meet in person with remote staff connected virtually. These Focused Forums are for staff only (MS and PhD levels). The lead for each monthly session rotates among staff. The format and content is at the discretion of the lead but can consist of a presentation on an analysis problem they want help with, an interesting project they worked on, a particularly useful software package, a journal article review, or a statistical method. During the last half hour, everyone provides a quick update of a project they are working on. MS-level staff have a separate meeting approximately monthly where they collaborate on various initiatives (e.g., development of standard operating procedures) and connect on topics germane to their positions. Administrative meetings with all staff and faculty area held quarterly. Rapid Relays or Spotlight Segments are commonly integrated into these meetings with a staff member presenting on a technical topic. Technical resources are maintained on a Microsoft Teams site.

**Table 3.** Case study 2: Quantitative Sciences Unit, Stanford University

Personnel	The Quantitative Sciences Unit (QSU) at Stanford University consists of 12 master’s level data scientists, 14 PhD-level data scientists, 4 administrative staff, and 7 faculty. Four staff (2 MS, 1 PhD, and 1 admin) are fully remote with the remainder on a hybrid schedule.
Goals and objectives	Continued learning from the group on data science methods but particularly on team science methods, as it’s the latter where there are challenges, especially regarding the collaborative process (e.g., do we always have to have an SAP in place that is preregistered; when can we deviate from SAP without creating a new one; what is the best practice for sharing data; what is the best way to disseminate findings to the collaborative team; or, how best to conduct reproducibility exercises).
Challenges	It can be challenging to have everyone engaged (junior members are quieter), and it is common for the same subgroup of individuals to contribute to the conversation. It is only the annual retreat where members of the group are required to be on site. Thus, many sessions require a hybrid meeting style. This style has worked okay but is less ideal than having everyone on site as during the pre-COVID era.
Activities in practice	The QSU primarily exploits and builds on the Focused Forum format to customize professional development activities. The team meets weekly for 2 hours in a brown bag session, where training and learning take place (troubleshooting on current collaborative challenges, training on team science principles, journal club format, and reviews of statistical analysis plans and nearly final manuscripts) and weekly for an informal lunch-and-learn meeting (informal get together where people troubleshoot on current projects from coding to modeling to other types of collaborative issues). Additionally, the QSU holds an annual 2-day retreat (1 day of wellness and career development and the other day a deep-dive training on one topic), and quarterly mini-retreats (1/2 day). Personnel heavily use a Slack channel for conversations on statistical and coding topics. The QSU also holds quarterly meetings with only the junior members, only the senior members (which includes the faculty), and monthly faculty meetings. Finally, the QSU hosts a forum called the Quantitative Sciences Unit Research Methods Seminar where the latest methods are discussed for the clinical and translational research community.

The UC Davis Biostatistics Support Unit is small and predominantly composed of staff (Table 2). Rapid Relays and Spotlight Segments have been incorporated into quarterly administrative meetings when salient topics arise. Monthly professional development meetings (Focused Forums) with just staff are also held. The lead for these meetings rotates among staff, and the lead determines the topic and format. These activities require little administration and financial support yet have been effective for knowledge transfer and community building.

The Stanford Quantitative Sciences Unit represents a large unit comprised of faculty and staff (Table 3). Their professional development activities are more structured and extensive than UC Davis with longer and more frequent meetings, retreats, and quarterly subgroup meetings for development activities customized to the diverse needs of unit members. These extensive activities necessarily require more substantial time and monetary resources and were made possible by strong Quantitative Sciences Unit leadership obtaining institutional backing.

Teams should experiment with different formats and schedules and be willing to adjust to changing needs, interests, and constraints. There is not a one-size-fits-all approach to professional development for collaborative biostatisticians or biostatistical units. The best approach is one that is cooperatively developed, sustainable, and flexible to the changing needs of the participants.

Biostatisticians who are interested in participating in professional development but whose units do not currently have any activities have different options depending on their organizational structure. Those working in larger biostatistical teams can use skills in “managing up” to work with their manager or group leader to incorporate new intra-unit professional development in team-level meetings [14]. Options are different when the manager/group leader is not amenable to incorporating professional development activities for the unit or for biostatisticians who do not work with a larger biostatistics group (e.g., isolated statisticians working in a clinical department). Some proposed internal professional development activities are possible to complete alone (e.g., learning how to implement and interpret results from a new statistical method), but most activities are more effective with a partner or small group. Biostatisticians within a unit can form their own professional development program in ways that do not conflict with regular working hours such as lunchtime sessions. For isolated biostatisticians, we recommend identifying another collaborative biostatistician at the institution who is interested in working together on common professional development goals. Others have recommended connecting with an isolated statistician at another institution through a network such as the American Statistical Association’s Statistical Consulting Section networking groups [15].

## Overcoming Obstacles to Success

A primary obstacle to engaging in intra-unit professional development activities is the high workload and insufficient staffing. Time constraints and competing priorities impede the ability of managers to initiate professional development programs as well as the ability of staff to meaningfully participate. Rapid Relays and Spotlight Segments integrated into regular staff meetings can be an effective strategy for time-crunched analysts.

Funding models of biostatical units are a consideration as well if the perception is that staff who are 100% funded on projects devote all working hours to these projects. In fact, such staff need to engage in various activities not directly related to projects such as required trainings and paid time off, the costs of which are shared among funders. Professional development should be viewed from this perspective (i.e., required training) and can be specifically identified as an activity covered by a funding agreement. Managers can emphasize to funders the necessity and value of professional development for technical staff. For more extensive trainings, managers of these units should use their position to advocate for institutional funding (i.e., “hard money”) to provide protected time for professional development for their staff members. Others have described the benefits of institutional “hard money” for biostatistical units [1] and have shown that institutional funding spent on biostatistical units have a high return on investment [16].

Biostatistics staff may not feel empowered to pursue professional development either individually or in concert with other staff. The manager plays an essential role in establishing the value of and creating an environment supportive of professional development. Managers can promote an expectation for professional development and provide protected time for activities through various avenues including establishing regular meetings specifically focused on professional development (Tables 2 and 3) as well as through annual performance reviews and goal setting. Managers can also consider developing an incentive structure for professional development activities. For example, a continuing education credit system could be developed where biostatistics staff receive credits for completing professional development activities. Depending on the comfort level of the biostatistician, professional development goals can increase in complexity over time, from delivering Rapid Relays or Focused Forums to participating in Extended Engagements. Managers do not need to shoulder the burden of developing intra-unit professional development programs on their own. Indeed, a more successful approach is to create a culture that provides time and value for ongoing professional development so that biostatistics staff members are empowered and encouraged to seek out and develop their own professional development opportunities.

The COVID-19 pandemic resulted in many collaborative biostatisticians working remotely. For teams working in a centralized location prior to the COVID-19 pandemic, staff could easily interact directly with other statisticians which supported ongoing learning and growth. With the rise of remote work, spontaneous interactions are less common, and engaging with another statistician has to be scheduled and purposeful. Routine meetings can help address this obstacle. Weekly 15-minute check-ins or longer, more structured meetings at less frequent durations can create the framework for professional exchanges. When intra-unit professional development activities occur virtually, sessions can be recorded and accompanying materials can be maintained in a central repository for future access by new or current staff.

## Discussion

Effective and successful collaborative biostatisticians have strong statistical skills in conjunction with well-honed interpersonal abilities, communication skills, and familiarity with biomedical topics. Becoming a proficient collaborative biostatistician necessitates continued development and improvement in both technical and nontechnical skills. Collaborative statisticians, both staff and faculty, are a critical workforce at academic medical centers, but their skill sets will need continuing refinement for them to be effective contributors in fulfilling their institution’s research missions.

In this paper, we emphasized the importance and value of making a structured and visible commitment to continued professional development of collaborative biostatisticians. We detailed intra-unit activities that can be integrated into routine operations for time and cost-efficient professional development. Through two case studies, we illustrated how to combine components and structure programs to align with an individual unit’s culture and setting.

Intra-unit professional development programs offer many benefits beyond their time and cost effectiveness. First, intra-unit programs can be tailored specifically to the needs and interests of unit members. Second and significantly, the benefits of an intra-unit professional development program are multiplicative, extending beyond improved skills and competencies. Junior and early career members can gain confidence in communicating with others, a vital skill for collaborative biostatisticians. Finally, group activities can increase camaraderie among members, build connections, and create a sense of belonging to a larger entity with a shared mission, factors that are important for job satisfaction and staff retention [9,17].

Intra-unit professional activities are complementary to and expand on traditional extra-unit activities and formal training (Fig. 1). Traditional extra-unit activities, which may be intra- or extramural, are generally longer duration activities, require commitment by an individual, may require financial support, and typically only serve one or a few individuals. Formal trainings typically have structured curricula that require considerable effort to develop and target a specific skill. While these activities will remain important, intra-unit activities offer opportunities for frequent, short, and diverse activities reaching many members in addition to more extensive intra-unit engagements that can rival traditional courses and workshops. Notably, ongoing mentoring from intra- and extra-unit mentors is an important source of professional development for members at all levels.

There are of course limitations and obstacles to instituting an intra-unit professional development program. While intra-unit activities are time- and cost-efficient relative to extramural activities, they still require a time commitment to organize, participate in, and maintain. Workloads as well as other professional activities (e.g., required training) for both managers and staff can interfere with maintenance and effectiveness of these programs. Managers have a vital role in sustaining professional activities, but some aspects of the professional development program can be delegated to other staff with the added benefit of developing their leadership and management skills.

Through these professional development activities, staff will be exposed to new ideas, collaborative approaches, and statistical methods as well as learn of supportive resources that they can draw on in the future. To enhance uptake of new skills, managers can encourage the application of new skills to specific projects as appropriate. Managers also have a responsibility to help unit members manage and prioritize project work and professional development activities and can do so by directing the use of development activities either as part of project work (e.g., researching a method to use) or separately.

Scheduling can be difficult for large units where members have conflicting meetings. Intra-unit activities can also be limited by skill sets and expertise of the members in the unit. However, this limitation can be overcome by inviting biostatistics experts to deliver focused technical seminars or clinical collaborators to introduce clinical subject matter. Also, many institutions have learning and development departments that will conduct workshops on nontechnical abilities for specific units. Finally, with the ease of holding virtual meetings, experts from other institutions can be recruited to deliver a seminar or training for individual units or multiple institutional units.

We believe a key to the success of these programs is establishing a culture and expectation of continued professional development from top-level managers and faculty to newly hired graduates. Managers can emphasize the importance of professional development by creating the structure for such efforts, dedicating resources, and integrating development goals into annual performance assessments and goal setting. Unit members should advocate for developmental activities on topics of interest to them, organize, and lead activities but most importantly actively participate in ongoing development.

Our suggestions for initiating and constructing an intra-unit professional development program are just a first step. Methods for evaluating and measuring the impact of these programs are largely undeveloped yet such metrics are important for demonstrating their value and continually improving their effectiveness. In the absence of metrics, managers have options for qualitatively assessing the effectiveness of the programs. Managers can regularly ask staff either in-person or through a survey if they find the activities to be valuable, if and how they have applied skills and techniques introduced at professional development activities, and how to modify the program to enhance learning and development.

Programs will also benefit from maintenance of professional development materials (e.g., slide decks, code examples, and references) in a searchable digital format. Use of these materials can be enhanced by managers regularly reminding unit members, particularly new members, of the availability of these resources. Recommendations for platforms and organizational structures of digital resources are currently lacking and will likely rapidly evolve given the proliferation of networking and digital storage options.

We emphasize that there is not a “one-size-fits-all” professional development program that will optimally meet the needs of all biostatistical units. Biostatistical units vary greatly in terms of the number of members, physical location of members (on-site at one location, in-person at multiple locations, hybrid, and fully remote), technical expertise, professional experience, funding, workload, and faculty engagement. As such, the best cadence, format, structure, and content of an intra-unit professional development program will vary. We have detailed several different formats for professional development activities. These components can be flexibly combined to create an appropriately scaled, customized program that is responsive to the needs and constraints of a particular unit. Ultimately though, the level and sustained success of an intra-unit professional development program will depend on the people involved and leadership endorsement. The strongest, longest lasting, and most effective programs will arise from a joint effort and commitment of time and resources by leadership, managers, and staff, with periodic reassessment and program modifications to meet changing unit circumstances and objectives.

## Supporting information

Taylor et al. supplementary material Taylor et al. supplementary material

## Supplementary material

The supplementary material for this article can be found at https://doi.org/10.1017/cts.2023.653.
