# Supplementary material for: Building a strong collaborative biostatistics workforce: Strategies for effective intra-unit professional development activities
Source: J Clin Transl Sci. 2023 Oct 19;7(1):e230. doi: 10.1017/cts.2023.653 (PMC10643907; doi:10.1017/cts.2023.653)
Supplement: Taylor et al. supplementary material [file S2059866123006532sup001.pdf]

SUPPLEMENTAL MATERIAL: ASSEMBLING THE COMPONENTS

We consider a hypothetical biostatistics unit consisting of 4 faculty and 12 staff (4 PhD, 8 MS level). Below are possible responses to questions on the Goals and Objectives for Professional Development Activities, the Resources and Environment, and Logistical Considerations sections. Based on these, we suggest an option for initiating and maturing a professional development program using our proposed components.

Question	Example response	Program consideration
Goals and Objectives for Professional Development Activities		
1. Who wants to participate in professional development? For those who do not, why not?	Staff want to participate but faculty are concerned about time commitment and value.	Focus initial activities on staff with invitation to faculty. Consider mix of short (e.g., Rapid Relays, Spotlight Segments) formats, possibly integrated with regular unit meetings and separate longer (e.g., Focused Forums) formats.
2. What professional development opportunities do unit members currently participate in?	Staff and faculty periodically attend conferences or seminars. Individual staff occasionally take external short courses.	Neither staff nor faculty are getting routine on-going development highlighting the value of an internal program.
3. What do unit members see as the unit's current professional development needs?	Staff members generally want to increase statistical methodological understanding and coding skills. Staff manager sees need for improving communication, collaboration and time management skills. Faculty are interested in negotiation, staff/student management and career navigation.	Work collaboratively to develop topic list and schedule. Staff manager may suggest topics for staff to consider.
4. If the unit includes faculty, are there differences in what types of professional activities staff and faculty want?	Staff are primarily interested in statistical knowledge and skills and want hands on activities. Faculty have greater interest in areas such as effectively managing staff/graduate students, negotiating budgets and conflict resolution with PIs.	Consider if a mix of joint staff and faculty and separate activities is needed. If there are other avenues for faculty professional development such as departmental or institutional sponsored activities, consider emphasizing staff needs in the initial professional development program.

Question	Example response	Program consideration
5. Are there subgroups (e.g., staff, junior faculty) who desire separate activities to address unique needs?	Based on responses to the previous question, it is apparent that staff and faculty have at least some different development interests. Staff also expressed interest in staff only meetings.	Consider a mix of joint staff and faculty and separate activities.
6. What knowledge, skills and abilities do unit members want to improve or acquire?	Staff members generally want to increase statistical methodological understanding and coding skills and manager sees need for improving communication, collaboration and time management skills but without specific topics identified initially.	Collaboratively brainstorm and rank specific topics to create a working "syllabus."
Resources and Environment		
7. How large is the team that will regularly participate in these activities?	Only staff can be expected to regularly participate.	Consider focusing initial development program on staff with invitation extended to faculty.
8. Do you currently meet regularly as a team? If so, how often do you meet and for how long?	Staff and unit manager currently meet bi-weekly for ~ 30 minutes with quarterly full unit meetings (staff and faculty) of 30-60 minutes	Existing bi-weekly meetings could support addition of a Rapid Relay. Existing quarterly meetings could support addition of a Spotlight Segment. Separate professional development meetings would be needed to accommodate longer-form activities such as Focused Forums or Extended Engagements.

Question	Example response	Program consideration
9. What is the typical focus of and reason for your existing team meetings?	Administrative	Consider possibility of adding professional development items to existing meetings (e.g., Rapid Relays)
10. Who attends the team meetings? Is it staff only or are faculty members in attendance as well?	See above	Professional development topics covered and duration of meeting may differ if both staff and faculty attend team meetings as opposed to just staff due to different interests and time constraints.
11. Realistically, to what extent can schedules and workload accommodate additional or expanded meetings?	Faculty and to some extent senior staff are hesitant to increase meeting load. Junior staff have more flexibility and their active participation is encouraged by management.	Existing schedules and workload appear able to accommodate a recurring but not too frequent professional development activity.
12. Given the goals and needs previously identified, what components can be used to meet the goals?	All of the components can be used.	While all components can be used, consider starting "small" with Rapid Relays and building from success.
13. Can components (e.g., Rapid Relays, Spotlight Segments) be combined with existing meeting structure?	Yes	Rapid Relays can easily be incorporated into bi-weekly staff meetings and quarterly unit meetings. A Spotlight Segment could be added to quarterly unit meetings.
14. Are professional development meetings separate from administrative meetings necessary and desirable?	Separate professional development meetings are desirable to provide more in-depth coverage of a topic and allow flexible participation.	Start with a monthly or quarterly Focused Forum

Question	Example response	Program consideration
15. Are financial resources necessary and available for any activities?	In general, financial to support travel, purchase of materials/supplies or conference registration will not be needed initially but some financial resources are available.	Consider developing a policy establishing time costs for professional development as part of required training and therefore as activities covered by external funding.
16. How much time do unit members have to attend and prepare for professional development activities?	Varies depending on person and short-term workload fluctuations but staff endorse their ability to contribute and participate on a regular basis.	Plan in advance. Rotate responsibilities among unit members. Impose a time limit for preparation.
Logistical Considerations		
17. How often do you want to meet and for how long?	Staff favor adding a once-a-month meeting focused on professional development separate from administrative meetings. Faculty do not want meetings more frequent than 1x month. Some staff are interested in longer term study sessions (e.g., Extended Engagements) but without a specific topic or leadership identified.	Consider starting with a once a month 1-hour meeting. Manager can consider encouraging and facilitating initiation of an extended engagement, perhaps leading an initial session.
18. Who will be in charge of deciding on topics and leading meetings? Will leadership rotate?	To reduce the individual burden, unit members agree to rotate leadership.	Manager or their delegate will schedule and remind topic leads.
19. What format will be used (e.g., in person, fully remote or hybrid)?	In general, meetings with be remote or hybrid to accommodate fully remote employees and to enhance participation.	

Question	Example response	Program consideration
20. What constraints or challenges are present, and how will these challenges be addressed?	With rotating leadership, quality of topic choice could vary which may reduce impact of the program.<br><br>Workload fluctuations could impede participation or a designated lead developing materials for their assigned date.	Consider short- and long-term objectives of program and developing a more formal syllabus.<br><br>Remind staff/faculty of up-coming commitments to lead a session. Check in early with leads and adjust.
21. Will resources developed as part of professional development activities be stored in a central repository for later access? If so, where will they be stored?	Unit members see value in retaining resources in a central repository.	Establish a central repository (e.g., Teams Site). Rotate management of the site yearly among unit members.

Based on the collective responses from staff and faculty for this unit, an initial professional development program could consist of a monthly Focused Forum and addition of Rapid Relays to both the bi-weekly and quarterly meetings when a salient topic arose. The monthly Focused Forum could be held over the lunch period to minimize work conflict. The lead could be rotated among staff and faculty with the lead for each session deciding on the topic and leading the session. Leads could be scheduled 6 to 12 months out to provide ample time for preparation or rescheduling. Slide decks, code or other resources would be maintained on a Team Site. This type of program could be implemented quickly and cost effectively. Although the program would not fulfill all of unit's professional development needs and interests, it would contribute to some objectives while working within time and workload constraints of participants and organizers.

The program could grow and evolve in a number of ways from this initial structure and organization. First, staff expressed interest in Extended Engagements. The unit manager could work with interested staff to identify a topic for an Extended Engagement, provide guidance on its organization and set expectations in terms of duration and outcomes. Second, the initial approach for the Focused Forum is unstructured in terms of topics covered. As noted in the table as considerations to responses to question 6, the full unit or a subset of members could develop a syllabus of related topics to be covered in a few sequential monthly sessions to support more targeted development. Implementing such a program would be more challenging and time-consuming given the need find people internally or externally to create sessions on specific topics. Such an endeavor could however start small. For example, a specific three-part topical series could be scheduled into the regular monthly meeting. Third, given the different professional development interests and constraints between staff and faculty, separate professional development activities targeting for faculty should be instituted. These could be quarterly meetings to minimize meeting burden and potentially take advantage of institutional or external offerings (e.g., webinars produced by the Association of Clinical and Translational Statisticians) to reduce material development burden.

When mature, the professional development activities for this unit would consist of:

1. Integration of Rapid Relays into regular administrative meetings
2. On-going monthly Focused Forums consisting of a mix of unit member selected and lead topics and short topical sequences to target specific development needs of the unit
3. Quarterly faculty-centric meetings/activities
4. Periodic Extended Engagements for staff

Over time the components and focus of the components could evolve in response to changing unit size, composition, and development needs.
